# Responses of Aspen Leaves to Heatflecks: Both Damaging and Non-Damaging Rapid Temperature Excursions Reduce Photosynthesis

**DOI:** 10.3390/plants8060145

**Published:** 2019-05-30

**Authors:** Katja Hüve, Irina Bichele, Hedi Kaldmäe, Bahtijor Rasulov, Fernando Valladares, Ülo Niinemets

**Affiliations:** 1 Institute of Agricultural and Environmental Sciences, Estonian University of Life Sciences, Kreutzwaldi 1, 51006 Tartu, Estonia; shueve@gmx.de (K.H.); hedi.kaldmae@emu.ee (H.K.); bahtijor@ut.ee (B.R.); 2 Institute of Physics, University of Tartu, W. Ostwaldi 1, 50411 Tartu, Estonia; irina.bichele@ut.ee; 3 Museo Nacional de Ciencias Naturales, C.S.I.C., Serrano 115 dpdo, E-28006 Madrid, Spain; valladares@ccma.csic.es; 4 Estonian Academy of Sciences, Kohtu 6, 10130 Tallinn, Estonia

**Keywords:** hybrid aspen, heat stress, leaf temperature, photosynthesis inhibition, photosystem II, *Populus*, stomatal conductance, sunflecks

## Abstract

During exposure to direct sunlight, leaf temperature increases rapidly and can reach values well above air temperature in temperate forest understories, especially when transpiration is limited due to drought stress, but the physiological effects of such high-temperature events are imperfectly understood. To gain insight into leaf temperature changes in the field and the effects of temperature variation on plant photosynthetic processes, we studied leaf temperature dynamics under field conditions in European aspen (*Populus tremula* L.) and under nursery conditions in hybrid aspen (*P. tremula* × *P. tremuloides* Michaux), and further investigated the heat response of photosynthetic activity in hybrid aspen leaves under laboratory conditions. To simulate the complex fluctuating temperature environment in the field, intact, attached leaves were subjected to short temperature increases (“heat pulses”) of varying duration over the temperature range of 30 °C–53 °C either under constant light intensity or by simultaneously raising the light intensity from 600 μmol m^−2^ s^−1^ to 1000 μmol m^−2^ s^−1^ during the heat pulse. On a warm summer day, leaf temperatures of up to 44 °C were measured in aspen leaves growing in the hemiboreal climate of Estonia. Laboratory experiments demonstrated that a moderate heat pulse of 2 min and up to 44 °C resulted in a reversible decrease of photosynthesis. The decrease in photosynthesis resulted from a combination of suppression of photosynthesis directly caused by the heat pulse and a further decrease, for a time period of 10–40 min after the heat pulse, caused by subsequent transient stomatal closure and delayed recovery of photosystem II (PSII) quantum yield. Longer and hotter heat pulses resulted in sustained inhibition of photosynthesis, primarily due to reduced PSII activity. However, cellular damage as indicated by increased membrane conductivity was not found below 50 °C. These data demonstrate that aspen is remarkably resistant to short-term heat pulses that are frequent under strongly fluctuating light regimes. Although the heat pulses did not result in cellular damage, heatflecks can significantly reduce the whole plant carbon gain in the field due to the delayed photosynthetic recovery after the heat pulse.

## 1. Introduction

Plant leaves are exposed to strong fluctuations in radiation and temperature in the field [1,2,3,4]. Leaf assimilation rates decline at suboptimal illumination as well as at supraoptimal illumination or temperature. Especially within and beneath the forest canopy, sunflecks are an important energy source for plants [4,5] but even open-grown plants are exposed to strongly varying light conditions in days with intermittent cloud cover. Apart from providing photosynthetic energy, sudden exposure to direct sunlight can lead to considerable increases in leaf temperature [6,7] and photoinhibition [8]. In addition, leaf temperatures can rapidly change in variable wind conditions [9,10]. While a large number of studies have looked at photoinhibition that can be particularly severe when other environmental stresses interact with high light [11,12,13,14], leaf responses to heat pulses caused by direct sunlight and windy conditions alternated with calm periods have been studied less. This is a significant omission as in addition to diurnal variations in air temperature, leaf temperatures can increase rapidly during exposure to direct sunlight, rising to values more than 10 °C above the air temperature during exposure of shaded leaves to full sunlight [15,16], especially in conditions with little air movement and/or when the water supply of the plant is restricted, thus limiting cooling by transpiration [17]. Such high-temperature events, during which leaf temperatures may reach even 50 °C can importantly influence growth and ecological distribution of tree species [18]. A global assessment of tree mortality caused by drought and/or high temperature demonstrates that different forest types and climatic zones worldwide are vulnerable to dieback due to severe heat and drought stress [19]. Although heat stress is traditionally not considered a significant stress factor in Northern hemisphere hemiboreal ecosystems, tree mortality associated with heat waves and drought is not only restricted to the dry and hot biomes but threatens a much wider range of ecosystems [19].

The decrease in leaf assimilation rates at high temperatures can result from stomatal closure and inhibition of light and dark reactions of photosynthesis. There is a debate about the processes responsible for the heat-driven photosynthetic decline and about exact temperature thresholds at which high-temperature reduction of photosynthesis becomes irreversible [16,20,21,22,23,24,25,26]. Several authors consider the heat-dependent reduction of ribulose-1,5-bisphosphate carboxylase/oxygenase (Rubisco) activity due to impaired activity of Rubisco activase to be the primary determinant of heat-dependent reduction in photosynthesis [20,21,22]. Rubisco-dependent heat inactivation is thought to be fully reversible [20,21,22]. In contrast, earlier studies have suggested that sustained inhibition of photosynthesis in heat-stressed leaves is linked to reduced photosynthetic electron transport activity [27,28,29]. In fact, impairment of thylakoid reactions first, followed by Rubisco inactivation has been found to be responsible for the reduction in photosynthesis rate in heat-stressed leaves in the field [16,30,31]. In addition, severe heat stress can further lead to damage of photosynthetic machinery, including denaturation of membrane proteins and impairment of lipid-protein interactions [32,33,34], and time-dependent reductions in photosynthetic activity even after return to lower temperatures [24,25]. When the threshold for thermal damage of photosynthesis is exceeded, the rate of photosynthesis recovers slowly if at all due to the elicitation of programmed cell death like processes or necrosis [24,35,36].

The objectives of this study were two-fold. First, we intended to investigate the variation in leaf temperature under natural field conditions in hemiboreal trees to assess the potential significance of field variations in leaf temperature on leaf photosynthetic apparatus. Leaf temperatures were measured in field-grown shade-intolerant European aspen (*Populus tremula* L.) trees in hemiboreal Estonia and in hybrid aspen (*P. tremula* L. × *P. tremuloides* Michaux) saplings grown in a tree nursery. Second, we aimed at determining the kinetics of heat- and light-dependent changes in photosynthetic capacity under heat stress scenarios as may occur in the field. Under laboratory conditions, we applied heat pulses of different length to investigate the heat response of photosynthetic activity in the leaves of hybrid aspen. Due to its high growth rate, hybrid aspen is used in short-rotation plantation forestry for the production of pulpwood and it is widely grown in Scandinavian countries and in the Eastern USA. In Estonia, hybrid aspen plantations have been established since 1999; hybrid aspen has already shown to be a promising tree for pulpwood production especially on abandoned agricultural lands [37] and to be suitable for energy forestry for the production of biofuels [38]. 

The laboratory experiments were designed to assess the kinetics of heat- and light-dependent changes in photosynthetic capacity under heat stress scenarios simulating rapid environmental fluctuations in the field. To simulate the complex fluctuating temperature environment characteristic to the field environments, intact attached leaves were subjected to short temperature increases (“heat pulses”) of varying duration over the temperature range of 30–53 °C. Short-term rises of temperature lasting only a few minutes closely simulate the temperature environment in dense forest vegetation on sunny days [15]. During a sunfleck, the leaf is simultaneously exposed to high light and increasing temperature and therefore, in a second series of experiments, leaves were simultaneously exposed to high light intensity during the heat pulse. Our aim was to assess the degree of photosynthesis inhibition after a heat pulse over the physiological temperature range and determine the time needed for recovery.

## 2. Material and Methods

### 2.1. Assessment of Leaf Temperatures in the Field

The European aspen (*Populus tremula* L.) trees were investigated in a young (10–15-year-old) 10–12 m tall stand south of Tartu, Estonia (58°20’ N, 26°40’ E, elevation 70 m above sea level). The stand was relatively open with a leaf area index of about 3 m^2^ m^−2^ such that all leaves received full sunlight at least during some parts of the day. We monitored leaves at the edge of the forest that were exposed to full sunlight for most of the day as we expected that these leaves were potentially exposed to the most severe temperature stress [39]. The heatflecks are classically associated with understory plants but heatflecks do frequently also occur in open-grown leaves during intermittent cloud cover. Thus, the heatflecks can also significantly contribute to the variation in photosynthetic activity in shade-intolerant trees like aspen.

The meteorological data were obtained from the weather station at the Institute of Environmental Physics of the University of Tartu, which was ca. 2 km from the plant growth location (http://meteo.physic.ut.ee). At the time of the measurements in August 2007, the trees in the natural stand were severely water-stressed due to a drought period of 2 weeks preceding the measurements. The drought period was also combined with hot weather conditions for the first two weeks of August when the mean average air temperature was 20.5 °C (daily maximum 20–31 °C). This exceeded by 3.7 °C the long-term mean average air temperature for this region (16.8 °C for August according to the Estonian Weather Service, http://www.ilmateenistus.ee).

Hybrid aspen (*P. tremula* L. × *P. tremuloides* Michaux) clone No. CO5-99-34 (formerly clone 200, originating from Nurmijärvi in Finland) was studied at AS Plantex forest nursery located in Sillapää village, Räpina, Estonia (58°05’ N, 27°26’ E, elevation 40 m above sea level). The trees were well-watered in the nursery. The leaves were exposed to full sunlight and ambient conditions of wind, temperature, and relative humidity when the thermal images were taken. Additionally, hybrid aspen trees of the same clone obtained from AS Plantex Räpina forest nursery were grown in 3 L pots outdoors in Tartu, Estonia (58°21’ N, 26°42’ E). The height of the trees was about 50 cm at the time of the measurements. The trees were exposed to full sunlight and, if not noted otherwise, daily watered with tap water. 

To quantify the potential of leaf overheating during lightflecks in partly sunny days and leaf overheating in fully sunny days, thermal images of aspen leaves were taken with a FLIR B50 thermal camera (FLIR Systems Inc., Stockholm, Sweden) in representative days between 11:00–13:00 hours in August 2007. The emissivity of the leaves was set to 0.96.

### 2.2. Plant Material and Growth Conditions for Laboratory Experiments

Additional plants of *P. tremula* × *P. tremuloides* clone No. CO5-99-34 were grown in 3 L pots containing well-fertilized commercial soil mixed with sand (1:1) in a growth chamber. Light was provided by Osram metal halide lamps (Powerstar HQI-T 400 W/D daylight). Photosynthetic photon flux density was 300–400 μmol m^–2^ s^–1^ (14 hours light period, giving a daily photon flux density of 17.6 mol m^–2^ d^–1^, i.e., equivalent to light availability in partly clouded days in Estonian summer). During light/dark periods, the relative air humidity was 65/90% and the air temperature was 25/20 °C. The plants were 50 to 60 cm tall and fully-expanded mature leaves were used in all experiments. The laboratory experiments were conducted between 10:00–16:00 hours. The measurements were conducted in 12 replicate plants and altogether 68 individual leaves were measured (each individual leaf was heat-stressed only once). 

### 2.3. Experimental Set-Up

Part of an attached leaf was enclosed in a circular clip-on type leaf chamber of the two-channel fast-response measurement system (half-response time less than 3 s) optimized to measure fast transients of leaf gas-exchange [for full description see 40]. The temperature of the leaf chamber water jacket was controlled by a circulating water bath. The upper side of the leaf was glued to the glass window of the chamber water-jacket by starch gel to enhance the temperature exchange between the leaf and circulating water. In this system, the difference between leaf temperature and circulating water temperature was within 0.4 °C as measured by a thin copper-constantan thermocouple attached to the lower leaf surface. Gas exchange was measured only for the lower leaf surface where the stomata are found in aspen.

Synthetic air was mixed from pure N_2_, O_2,_ and CO_2_, and the air flow rate in the system was 0.5 mmol s^–1^. Chamber O_2_ concentration was kept at 21%, CO_2_ concentration at 360 μmol mol^−1^, and air humidity at 60%, except during heat pulses. CO_2_ concentration was monitored continuously with an infra-red CO_2_ analyzer LI-6251 (Li-Cor, Inc., Lincoln, NE, USA), and water vapor concentration by micro-psychrometers [40]. The leaf chamber could be illuminated by three Schott KL 1500 tungsten halogen light sources (Schott AG, Mainz, Germany) through a fiber optics light guide. One Schott light source equipped with a heat-reflecting filter (Optical Coating Laboratory, Inc., Santa Rosa, California, USA) provided white actinic illumination, the second light source was equipped with a 720 nm narrow-pass interference filter (Andover Corp., Salem, New Hampshire, USA) for far-red light (FR, 50 μmol m^−2^ s^−1^), and the third provided saturating pulses of white light of 11,000 μmol m^−2^ s^−1^ for chlorophyll fluorescence measurements. 

Chlorophyll fluorescence yields were measured with a PAM 101 fluorometer (Heinz Walz GmbH, Effeltrich, Germany) operated at 1.6 kHz pulse frequency for darkened leaves and 100 kHz for illuminated leaves and during saturating light pulses. After enclosure of the leaf in the gas-exchange cuvette, the leaf was kept in darkness for the first 20 min to obtain the dark respiration rate and chlorophyll fluorescence yields *F*_0_ and *F*_m_. For actinic illumination, we used white light with an intensity of 600 μmol m^−2^ s^–1^, if not noted otherwise. The illuminated leaf was kept at 25 °C and 360 μmol mol^–1^ CO_2_ until steady-state net assimilation and transpiration rates were reached, typically about 20 min after switching on the light. Steady-state fluorescence yield (*F*_s_) was measured continuously and saturating pulses of actinic light (11,000 μmol m^−2^ s^−1^) were used to measure the light-adapted maximum fluorescence yield *F*_m_´ and calculate the effective quantum yield of photosystem II (PSII, *Φ*_PSII_) as (*F*_m_´ − *F*_s_)/*F*_m_´. 

### 2.4. Temperature and Light Increase Experiments

**Figure 1:**
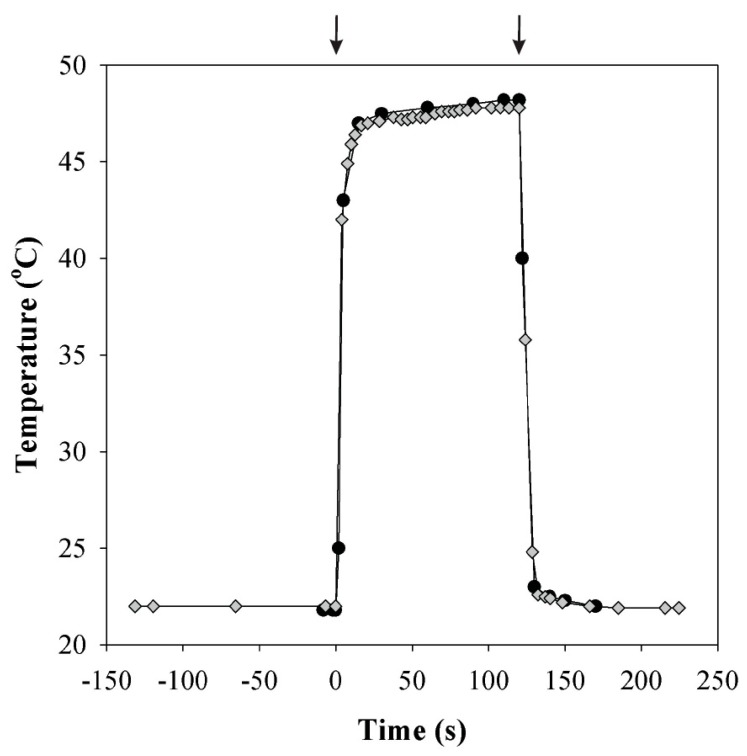
A representative illustration of leaf chamber temperature (black circles) and leaf temperature (grey diamonds) during a simulated heat pulse. The temperature of the leaf chamber was changed from 22 °C to 49 °C for 2 min using two temperature-controlled water baths. The leaf temperature remained slightly below the chamber temperature (by 0.4 °C in the experiment shown) due to leaf transpiration. Arrows indicate the beginning and end of the heat pulse.

To simulate rapid heat pulses resembling the dynamic leaf temperature environment in the field, we used two separate circulating water baths to adjust the temperature of water flowing through the leaf chamber jacket. Switching the water flow from one water bath to the other enabled a change in chamber temperature within 15 s (Figure 1). Depending on the leaf transpiration rate, the leaf temperature was at most 0.4 °C below the set temperature. Using this system, leaf temperature was increased rapidly for a short time spans of 2 to 5 min from 25 °C to temperatures between 33 and 49 °C (*n* = 25 independent leaves) or from 33 °C to temperatures between 40 and 53 °C (*n* = 25). After the heat pulse, leaf temperature was returned rapidly to the baseline temperature of 25 °C or 33 °C, as demonstrated in Figure 1.

In the second series of experiments, light intensity was increased to 1000 μmol m^−2^ s^−1^ 30 s before the heat pulse and returned to 600 μmol m^−2^ s^–1^ just after the heat pulse. Six replicate experiments under a constant light intensity of 600 μmol m^−2^ s^−1^ and six under 1000 μmol m^−2^ s^−1^ were performed. 

In all experiments, CO_2_ and H_2_O exchange rates and fluorescence yields were recorded continuously through the experiments. Before, during, and after the heat pulses, saturating pulses of white light were given in ca. 100 s intervals to monitor changes in *F*_m_´ and effective quantum yield of PSII.

### 2.5. Membrane Permeability Assays

Membrane permeability after heat treatment was determined by measuring electrolyte leakage from leaf tissues according to the established protocols [41,42,43]. Plasmalemma breakage releases ions into the water, increasing conductivity. Leaf disks of 0.5 cm diameter were wrapped in a thin polyethylene film and submerged in water at the desired temperature for 5 min. The controls were submerged in water at 25 °C. After the heat treatment, the disks were unwrapped and immersed in 5 mL deionized water and kept in darkness. The conductivity of the water was monitored by conductometer HandyLab LF1 (Schott Instruments, Germany) after 24 h to allow diffusion of leaf electrolytes from damaged cells into the water. After these measurements, the vessels with disks were boiled for 5 min, cooled to room temperature, and measured again to obtain the maximal conductivity of samples. The measured electrolyte leakage at any given treatment was expressed relative to the maximal conductivity obtained after boiling the sample. 

### 2.6. Data Analyses

Linear and non-linear regressions were used to analyze the relationships between the changes in foliage photosynthetic characteristics and heat pulse length and temperature during the heat pulse. Differences in the changes in foliage photosynthetic characteristics among heat pulse temperatures and among heat pulse lengths, and among different light intensities at given heat pulse temperatures and heat pulse lengths were separated by one-way analyses of variance (ANOVA) using R 3.5.3 with the standard packages [44]. All statistical relationships were considered significant at *p* < 0.05.

## 3. Results

### 3.1. Leaf Temperatures in the Field

**Figure 2:**
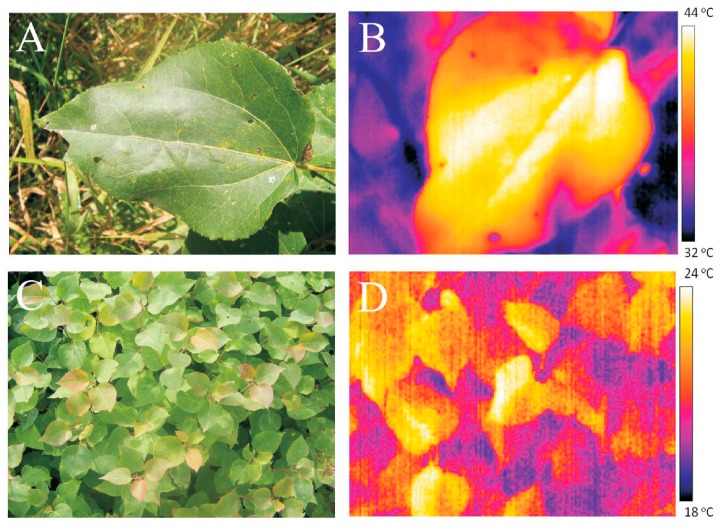
Leaf temperature under field conditions in European aspen (*Populus tremula*) in a naturally regenerated stand in the vicinity of Tartu, Estonia (**A**,**B**), and in hybrid aspen (*Populus tremula* × *P. tremuloides*) in Räpina, Estonia tree nursery (**C**,**D**) in representative summer days visualized by infra-red thermal images. Photographs in visible light on the left and infra-red thermal images on the right. The photographs were taken in mid-day. The *P. tremula* leaf was photographed on 13th of August 2007 when the air temperature raised to 30.7 °C, and that of hybrid aspen on 24th of August 2007 when the air temperature raised to 21 °C.

Leaf temperature in the field was dependent on weather conditions and plant water status, and it was heterogeneous within the leaf lamina. Maximum and minimum surface temperatures of the same leaf could differ by up to 10 °C among the leaf lamina areas positioned at slightly different angles relative to the sun (e.g., central vs. marginal areas), and among the same leaf regions during short time spans of a few minutes (Figure 2A,B for representative visible light and infra-red images for European aspen (*P. tremula*) and Figure 2C,D for hybrid aspen (*P. tremula* × *P. tremuloides*) leaves in the field). 

In a hot partly cloudy day (air temperature during the measurements of 29 °C, wind in gusts ≤3.2 m s^−1^), measurements in the drought-stressed trees of *P. tremula* at the edge of the forest demonstrated that leaf temperature was usually 1–2 °C above air temperature, while the maximum leaf temperature was on average 8 °C, while in extreme cases up to 15 °C above air temperature (Figure 2A,B). Under these rather extreme, but physiologically realistic conditions, leaf temperature varied between 26 and 44 °C (34–44 °C for the leaf depicted in Figure 2A,B) with an average ± SD of 34.1 ± 5.9 °C (*n* = 16). 

In contrast, in a sunny cooler day (air temperature during the measurements between 22.3 °C and 24.5 °C, wind in gusts, 3.8 m s^−1^), the leaf temperature of well-watered trees of hybrid aspen in the tree nursery varied between 21.5 °C and 26.5 °C (Figure 2B) with an average ± SD of 24.6 ± 2.0 °C (*n* = 35). Leaf temperatures of potted well-watered hybrid aspen trees growing in full sunlight were similar to those in the nursery trees (data not shown). However, when water was withheld for two weeks preceding the measurements, these plants exhibited the highest difference between leaf and air temperatures. In these drought-stressed plants, in a moderately warm day (air temperature 25 °C), leaf temperatures increased to 42–45 °C (average ± SD of 43.5 ± 1.0 °C, *n* = 20).

**Figure 3:**
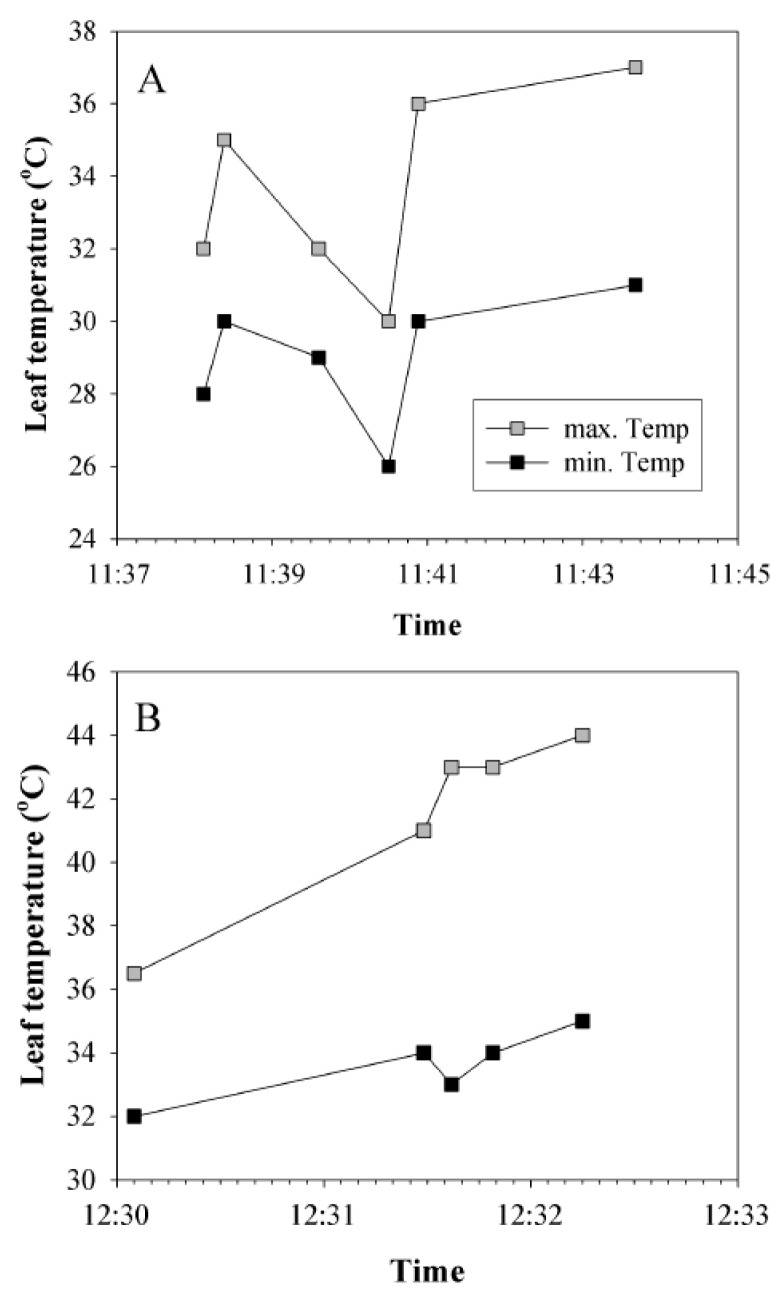
Characteristic alterations in leaf temperature in aspen leaves upon natural changes in exposure to sunlight due to clouds. Maximum temperature (grey squares) and minimum temperature (black squares) of the same leaves are indicated. (**A**): A leaf of a young potted (3 L pot) hybrid aspen (*P. tremula* × *P. tremuloides* clone No. CO5-99-34) tree measured on a partly cloudy moderately hot day (air temperature 25 °C). (**B**): A leaf of a European aspen (*P. tremula*) tree growing at the edge of a small forest south of Tartu, Estonia measured on a partly cloudy hot day (air temperature 29 °C).

Leaf temperature was further strongly variable in time, especially in partly cloudy days, and in days with variable wind speed. Illumination by direct sunlight led to a fast increase in leaf temperature, especially when there was no or little wind. An example is shown in Figure 3A. When direct sunlight was interrupted by a cloud, leaf temperature decreased (11:38–11:40). Exposure to direct sunlight at 11:40 induced a steep increase in leaf temperature by 4 °C (minimum increase in leaf temperature) to 6 °C (maximum increase in temperature) within 23 s. A slower increase, but to a much higher temperature is shown in Figure 3B, when a sudden exposure to full sunlight in calm conditions resulted in an increase of the maximum leaf temperature by 7.5 °C in 2 min and the leaf temperature reached 44 °C.

### 3.2. Responses of Photosynthesis to Heat Pulses Applied under Laboratory Conditions

**Figure 4:**
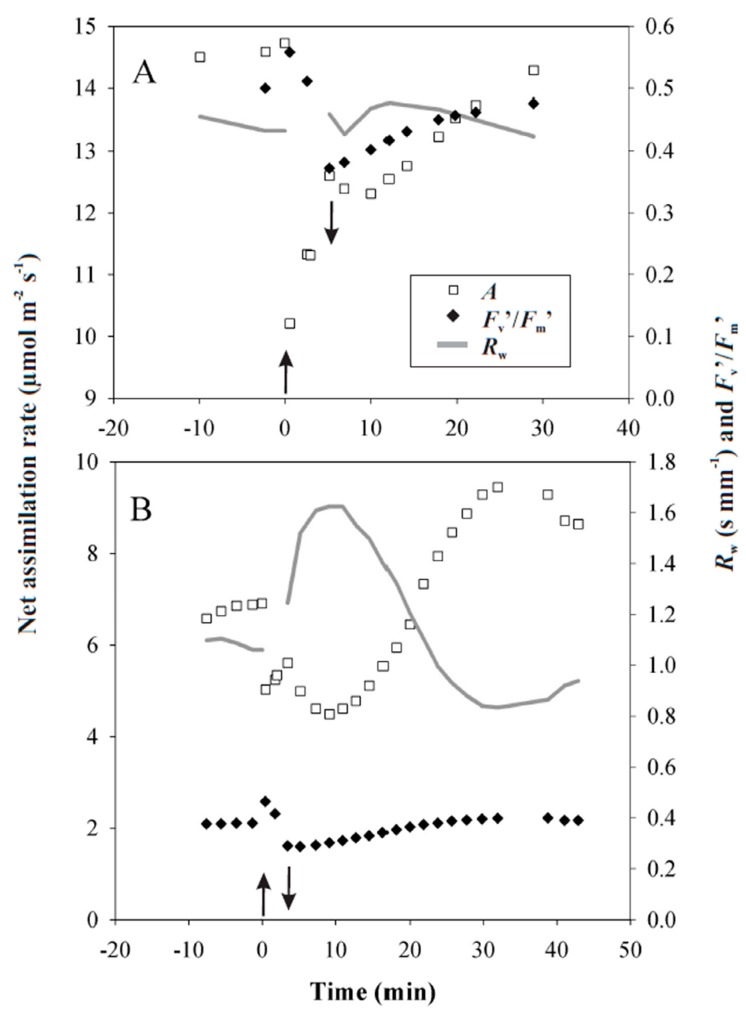
Representative responses of leaf CO_2_ uptake (*A*, open squares), effective quantum yield of photosystem II ((*F*_m_´ − *F*_s_)/*F*_m_´, black diamonds) and stomatal resistance to water vapor (*R*_w_, grey line, *R*_w_ is the inverse of stomatal conductance) to a simulated heat pulse in two different leaves of hybrid aspen (*P. tremula* × *P. tremuloides* clone No. CO5-99-34). Heat pulses were applied under laboratory conditions using the ultra-fast leaf gas-exchange apparatus (Figure 1 for the rate of change in leaf temperature). Leaf temperature was increased from 25 °C to 40 °C at time 0. For the first leaf, the temperature was raised for 3 min (**A**), for the second leaf for 2 min (**B**), and subsequently reduced again to 25 °C. Arrows indicate the beginning and the end of the heat pulses. Photosynthetic photon flux density was 600 μmol m^−2^ s^−1^. Data for *R*_w_ are not shown during the heat pulse, as the measurements were not reliable due to changes in relative humidity during the pulse.

Attached leaves were subjected to heat pulses of different duration and different temperatures under laboratory conditions. During moderately severe heat pulses, leaf net assimilation rate, *A*, and the effective quantum yield of PSII, *Φ*_PSII_, were somewhat enhanced, typically by 5–15% relative to the initial values (Figure 4 for representative experiments with heat pulses of 40 °C applied for 2 or 3 min). Right after the heat pulse when the leaves were returned again to 25 °C, both *A* (reduced to 85.5% of the initial value for the 2 min and to 81.2% for the 3 min heat pulse) and *Φ*_PSII_ (reduced to 74% for the 2 min and to 76% for the 3 min) were reduced (Figure 4A,B). Changes in stomatal resistance, *R*_w_, right after return to 25 °C were variable (mostly *R*_w_ increased but sometimes it decreased or was constant, Figure 4A,B). Both *A* and *Φ*_PSII_ started to recover after the heat pulse and almost fully recovered or reached even somewhat higher values after 30–40 min recovery. Most of the leaves reacted to the heat pulse with a transient closure of stomata (for example, Figure 4B), which transiently decreased *A* even more. 

**Figure 5:**
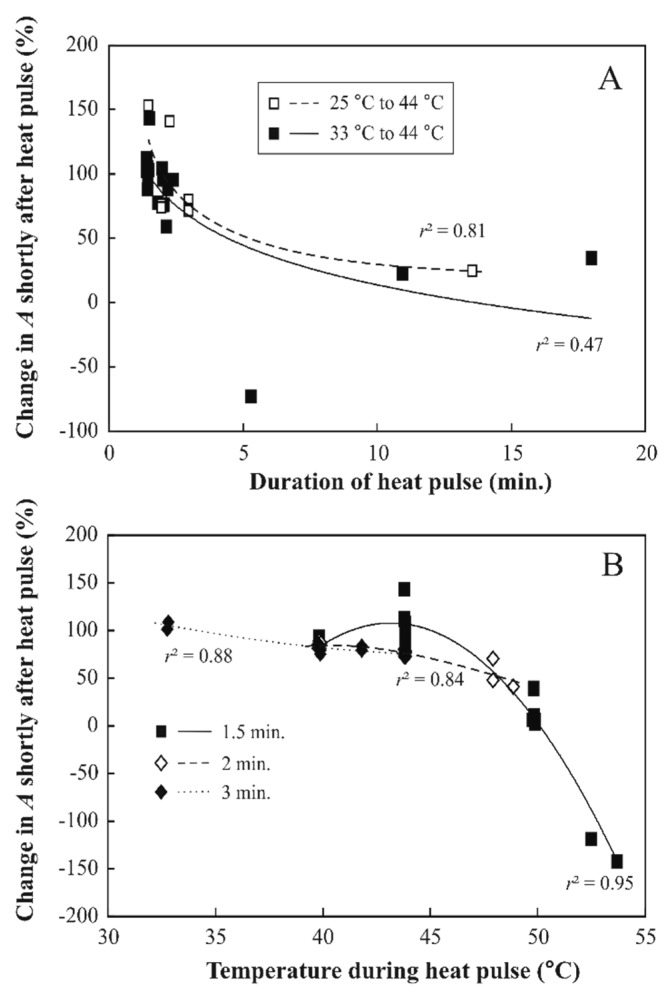
Changes in leaf net assimilation rate in dependence on the duration (**A**) and temperature (**B**) during the heat pulse in hybrid aspen (*P. tremula* × *P. tremuloides* clone No. CO5-99-34). In A, the effects of duration of heat pulse are compared among temperature changes 25 to 44 °C (*n* = 8 independent leaves) and 33 to 44 °C (*n* = 18). In B, the effects of heat pulses of 1.5 min (*n* = 22), 2 min (*n* = 11) and 3 min (*n* = 12) duration are compared. Net assimilation rate (*A*) was assessed after 30 s following the application of the heat pulse and is shown as % of *A* just before the heat pulse. Negative values indicate CO_2_ evolution, i.e., respiration after the heat pulse. In A, the data were fitted by non-linear regressions in the form *y* = *a*Log(*x*) + *b*. In B, the data were fitted by second-order polynomial regressions. All regressions are significant at *p* < 0.001.

The extent of photosynthesis inhibition immediately after the heat pulse depended on the temperature during the heat pulse as well as on the duration of the heat pulse (Figure 5). After the 44 °C heat pulses of 1.5–2 min, average (±SD) net assimilation rate relative to pre-treatment value was 95 ± 26% (for both 25 °C to 44 °C and 33 °C to 44 °C temperature treatments pooled, *n* = 15, Figure 5A). The large variability in changes in *A* after the heat pulse primarily reflected differences in how stomata responded to the heat pulse (see Figure 4 for contrasting patterns). This variability was especially large for the 1.5 min heat pulse (Figure 5B, average ± SD relative net assimilation rate after the 44 °C heat pulse of 1.5 min was 103 ± 15%, *n* = 12, but it was 75.6 ± 2.6% for 2 min and 73.9 ± 5.1% for 3 min heat pulse, *n* = 4 for both; the average values for both 2 min and 3 min heat pulses are lower than that for the heat pulse of 1.5 min, *p* < 0.005 according to one-way ANOVA). 

Ten minutes after the 1.5–2 min, 44 °C heat pulses, the inhibition of *A* had further decreased to 60–80% of the assimilation rate before the heat pulse, primarily due to stomatal closure. Nevertheless, leaf photosynthesis still recovered to pre-stress values after 30–40 min (data not shown). In the case of longer heat pulses, the recovery after 30–40 min was only partial (75–95% relative to the initial value, data not shown).

With increasing temperature during the heat pulse, the percentage of photosynthesis remaining first changed slowly up to a temperature of 44 °C then the decrease of photosynthetic reduction accelerated and starting from 50 °C the photosynthetic activity was close to zero (Figure 5B). For heat pulses up to a temperature of 44 °C and a duration of 2 to 3 min, *A* and *Φ*_PSII_ usually recovered to their former values (or even stabilized at somewhat higher values) after the heat pulses, but at higher temperatures, the recovery was only partial (Supplementary Figure S1 for a representative response to a heat pulse of 50 °C for 2 min). 

### 3.3. Heat Pulses Combined with Higher Light Intensity

An increase of light intensity from 600 μmol m^−2^ s^−1^ to 1000 μmol m^−2^ s^−1^ for 3 to 4 min simultaneously with heat pulses up to a temperature of 44 °C and a duration of 2 to 3 min did not affect CO_2_ uptake or *Φ*_PSII_ drastically. Both *A* and *Φ*_PSII_ were decreased by maximally 5% directly after the application of higher light and both recovered to their former values (or higher values) after 5 to 10 min. The effects of combinations of heat pulses with simultaneous application of high light of 1000 μmol m^−2^ s^−1^ on foliage photosynthetic characteristics were not significantly different from the effects of heat pulses applied at 600 μmol m^−2^ s^−1^ (data not shown; *p* > 0.1 for comparisons of reductions in *A* at given heat pulse lengths and temperatures). 

### 3.4. Changes in Plasmalemma Conductivity

**Figure 6:**
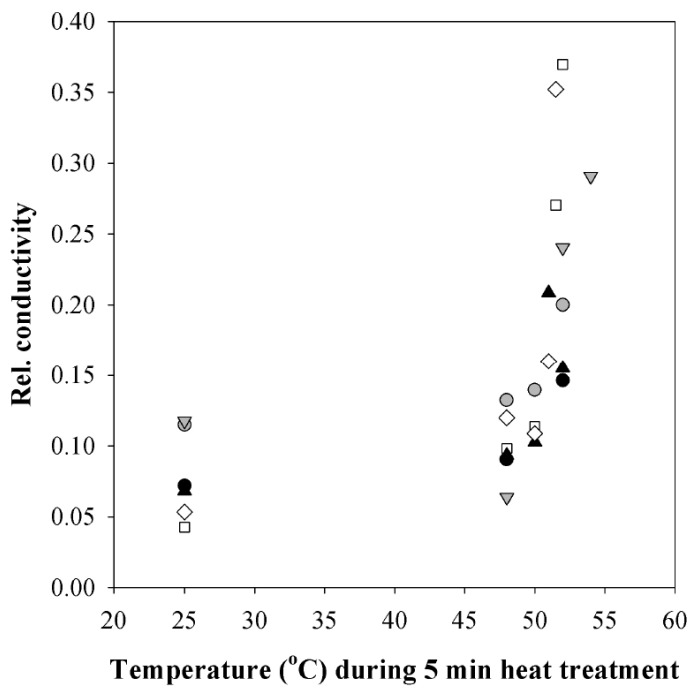
Relative conductivity of leaf disks after treatment to 5 min heat pulses of different temperature in hybrid aspen (*P. tremula* × *P. tremuloides* clone No. CO5-99-34). The relative conductivity is calculated as the conductivity after the treatment divided by the maximum conductivity obtained after boiling the leaf disk for 5 min. Different symbols correspond to different leaves and each symbol corresponds to a different leaf disk.

Relative conductivity of deionized water as changed by heat-treated leaf disks was used as a measure for plasmalemma intactness. Relative conductivity of leaf disks increased sharply at 51 °C (Figure 6) indicating the onset of major cellular damage.

## 4. Discussion

### 4.1. Leaf Temperatures in the Field

Based on observations of Singsaas et al. [15] and Valladares and Niinemets [2] as well as on measurements shown in this work, exposure of leaves to temperatures of 40–44 °C at least for a few minutes can be regarded as physiological conditions that can occur in the natural field environments. Our study demonstrates that even in the Northern hemisphere hemiboreal climate, such conditions can be experienced during sunflecks on moderately hot summer days with maximum air temperatures between 25–30 °C, especially in calm conditions ( 2,3 ) and when plant transpiration is restricted due to limited water availability.

Studies on the photosynthetic responses to sunflecks have been extensive [5] though mostly concerned with the influence of sudden high light on photosynthesis of shade-adapted leaves. High light intensity during a sunfleck may importantly enhance leaf carbon gain; however, longer lighflecks can also curb photosynthesis due to photoinhibition [45,46,47]. A sudden, strong illumination may further cause a rapid rise of the leaf temperature [7,48]. Such combined high light/high-temperature events are usually short and in the range of minutes. For instance, leaf temperatures of tree seedlings growing in a tropical rain forest were shown to be highly dynamic, whereas the peaks in leaf temperature depended on the length and intensity of sunflecks [7]. 

### 4.2. Reversible Changes in Photosynthetic Characteristics to Heat Pulses

The optimum temperature for net assimilation rate, *A*, at the current ambient CO_2_ concentration is around 30 °C or slightly above, while the optimum temperature for photosynthetic electron transport is somewhat higher [14,35,49]. Exposure to moderately elevated temperatures higher than the photosynthetic optimum, between 35–40 °C is typically fully reversible, but exposure to temperatures higher than 40 °C is often associated with a sustained reduction of net assimilation rates [50,51]. Furthermore, high leaf temperatures, especially in combination with high irradiation, can cause photoinhibition and leaf damage [14,52], and thus, it is essential to understand the natural variability in leaf temperature as well as the effects of these temperature variations on photosynthetic carbon gain and underlying partial photosynthetic limitations. As the damaging effect of heat on photosynthetic processes commonly scales with the integral of the heat sum above the threshold temperature, the critical heat dose [28,35,53], short-term heat pulses may not directly damage the leaf photosynthetic apparatus. However, heat may still result in a reduction in foliage photosynthetic rates due to temporal inhibition of dark or light reactions of photosynthesis or stomatal closure and the overall effect on daily carbon gain will depend on how fast foliage photosynthesis recovers after the exposure to the heat pulse.

In our experiments, short heat pulses with temperatures up to 44 °C immediately reduced *A* and the effective quantum yield of PSII, *Φ*_PSII_ (Figure 4 for representative kinetics and Figure 5 for general patterns). As stomatal response kinetics is on the order of minutes, the fast change in *A* right after the temperature change (Figure 4) cannot be explained by changes in stomatal openness. Such a rapid effect might reflect reductions in CO_2_ solubility in high temperature and increases of photorespiration. In addition, dark respiration can increase several-fold during a heat pulse [24,54]. On the other hand, the reduction in *Φ*_PSII_ also suggests that PSII could have been partly impaired as has been observed in leaves exposed to moderately high temperatures [55,56].

After the return to the lower temperature, *A* was further inhibited for a significant period of time, for 10–40 min (Figure 4A,B). Given that dark respiration recovers within a few minutes to its former value when the leaves are returned to the lower temperature [24], the sustained reduction in CO_2_ uptake after the heat pulse is not likely associated with enhanced respiration. In fact, the data indicate that the duration of inhibition was driven both by the recovery of *Φ*_PSII_ and changes in stomatal resistance. For instance, in Figure 4A, the photosynthetic inhibition after the heat pulse, between 2–30 min was primarily driven by reduced *Φ*_PSII_ and, to a lower extent, between 7–20 min by a moderate increase in stomatal resistance. It is plausible that *R*_w_ started to increase (stomata closed) as a response to the reduced net assimilation (and therefore rising internal CO_2_ concentrations) after the heat pulse. In addition, vapor pressure deficit also increased during the heat pulse, potentially further contributing to stomatal closure. In contrast, in Figure 4B, the photosynthetic inhibition between 2–15 min was primarily driven by the closure of stomata, a response that was much stronger than for the leaf in Figure 4A. However, in this case, the closure was followed by enhancement of stomatal openness and a greater level of *A* between 22–40 min than before the heat pulse. Rapid changes in environmental conditions can cause oscillations in *A*, *Φ*_PSII_, and *R*_w_ [39,57,58,59,60]. Leaf-to-leaf differences in the induction of oscillatory responses could have been responsible for leaf-to-leaf differences in the afterstress responses. Although the heat pulses of moderate severity did not lead to damage, the prolonged reduction of *A* most strongly contributes to the leaf carbon gain and such effects need to be considered in simulating daily leaf carbon uptake.

### 4.3. Irreversible Photosynthetic Changes to Longer and Hotter Heat Pulses 

A moderately severe heat pulse (up to 44 °C for up to 3 min) resulted in a fully reversible decrease of photosynthesis, while more severe heat pulses (more than 44 °C or 44 °C for longer periods) resulted in a sustained inhibition. The inhibition of photosynthetic processes was dose-dependent varying both with the temperature during the heat pulse and with the time the leaves were exposed to the high temperature (heat dose, Figure 5). The dose dependence observed in these experiments is in accordance with previous observations [28,53,61,62].

Hybrid aspen, although grown under artificial illumination in a plant growth chamber, proved remarkably resistant against heat pulses. While the temperatures reached during the heat pulses in the field are likely to cause transient decreases of photosynthesis, major cellular damage as indicated by increased conductivity was found only above 50 °C (Figure 6). Such high leaf temperatures can occur in the field on hot summer days, especially in water-stressed plants and in hotter biomes [17,18,63]. Remarkably, even after a heat pulse of 50 °C for 2 min, the treated leaf maintained a positive net CO_2_ assimilation (Figure S1), but the values of *A* and *Φ*_PSII_ were much lower than before the heat pulse and did not recover, indicating that the inhibition of CO_2_ assimilation was partly irreversible. Although leaf temperatures around 50 °C are unusual in hemiboreal and cool temperate climates, the dose-response of photosynthesis to heat stress implies that even exposure to supraoptimal temperatures observed in the field ( 2,3 ) can lead to irreversible or slowly reversible decreases in photosynthesis (Figure 5). 

Different mechanisms have been proposed for the decrease of CO_2_ uptake at high temperatures. A number of studies have suggested that PSII electron transport and oxygen-evolution are the most heat-sensitive processes [27,34,55,56,64,65]. In addition, heat stress has been shown to lead to enhanced thylakoid leakiness [16,55,56]; this together with reduced PSII electron transport rate ultimately limits the availability of ATP and NADPH and synthesis of ribulose 1,5-bisphosphate RubP [31,66]. Other studies have suggested that it is the reduction of Rubisco activity due to heat-sensitive Rubisco activase that is responsible for reduced photosynthesis under heat stress [20,21,22,65]. Yet, experimental data showing the presence of one limitation, either electron transport or Rubisco activity, do not rule out that the other limitation is also operative.

In fact, an increasing body of evidence suggests that a combination of inhibited electron transport and deactivation of Rubisco is responsible for the decrease of net CO_2_ uptake under heat stress. Inhibition of photosynthetic electron transport can be the first response, but as it leads to reduced RubP concentration, it can also lead to down-regulation of Rubisco activity in conditions when RubP is no longer saturating and the carbamylation status of Rubisco decreases [30,31]. This reasoning is supported by the evidence that RuBP level decreases already after a few seconds in high temperature when there is still no change in Rubisco activity [30]. Downregulation of Rubisco may be a protective response avoiding more deleterious effects on Rubisco and the whole photosynthetic machinery [23,67]. 

On the other hand, it has also been demonstrated that once the critical heat dose has been exceeded, there is a sustained or even progressive reduction in photosynthetic activity at lower temperatures as the result of propagation of reactive oxygen species and onset of programmed cell death like processes [24]. Tóth et al. [68] observed PSII damage and subsequent recovery after heat pulses of only 20–40 s at 50 °C. Leaves after this treatment showed heat stress symptoms like the loss of oxygen evolution activity and a strong decrease of *Φ*_PSII_ but they did not exhibit signs of visible damage. In a recovery period, damaged PSII was replaced by newly synthesized PSII. This recovery process, however, took about 48 h [68], much longer than the recovery observed in our experiments. Such a kind of damage may, for example, have occurred during the heat pulses at 50 °C (e.g., Figure S1), where only a partial recovery was observed during the experiment. We conclude that a combination of factors, operating in short and long term are responsible for decreased photosynthesis during and after heat pulses exceeding the threshold of physiological tolerance. 

### 4.4. Changes in Light Intensity during Heat Pulses Had Only A Minor Effect on Heat Resistance

Light has been reported to have ambiguous effects on leaf photosynthesis at elevated temperatures. Light may protect the photosynthetic system such that high-temperature treatment under light can cause less deleterious effects than the same temperature treatment in darkness. For example, Havaux et al. [69] observed that oxygen evolution and variable fluorescence at 40 °C were much more stable in the light than in the dark. High light, on the other hand, may induce photoinhibition, causing additional damage. Leakey et al. [7] reported that a temperature of 38 °C resulted in a larger inhibition of photosynthesis during a sequence of shade and sunflecks than under continuous illumination. In our experiments, the high light intensity of 1000 μmol m^−2^ s^−1^ was about three times higher than experienced by hybrid aspen plants during their growth in the growth chamber. However, high light during the heat pulse did not influence the inhibitory effect of the heat pulse, neither positively nor negatively, and nor did it influence the recovery time. In fact, net assimilation rate at 1000 μmol m^−2^ s^−1^ light was enhanced compared to 600 μmol m^−2^ s^−1^ light, indicating that 600 μmol m^−2^ s^−1^ was still a limiting light intensity. Thus, the leaves of hybrid aspen proved to be remarkably tolerant to the high light in our study, suggesting that the primary deleterious effect of rapid heating of leaves exposed to sudden changes in light is due to temperature not due to enhanced illumination.

## 5. Conclusions

Leaves under field conditions in the forest or at the forest edge experience highly dynamic incident light and temperature environments with light intensity varying more than an order of magnitude and leaf temperature more than 10 °C over short time periods. As the results of the current study demonstrate, rapid fluctuations in temperature may lead to a considerable inhibition of photosynthesis rates. In our experiments, we found that a short heat pulse, as may be caused by exposure of shaded leaves to direct sunlight in the field, caused partial and transient photosynthesis inhibition that may suppress photosynthetic carbon gain for the following half an hour. The temporal reduction of carbon gain following heatflecks can be highly significant for forest understory plants struggling at the verge of positive carbon balance. In addition, heat pulses exceeding the leaf physiological heat resistance can also occur under field conditions. These heat pulses cause irreversible inhibition of photosynthesis, resulting in sustained reduction of photosynthetic carbon gain. So far, such short-term heat effects on photosynthesis have found little consideration in forest stress models. As plant leaves are often exposed to temperatures of more than 5–10 °C above their photosynthetic temperature optimum during heatflecks in the field, heatfleck responses need to be incorporated in current physiological plant carbon gain models. 

## Supplementary Materials

The following are available online at https://www.mdpi.com/2223-7747/8/6/145/s1, Figure S1: A representative experiment showing changes in net assimilation rate (*A*, open squares) and effective quantum yield of Photosystem II (*F*_m_´−*F*_s_)/*F*_m_´ (black diamonds) in response to the increase of leaf temperature from 30 °C to 50 °C for 2 min in hybrid aspen (*P. tremula* × *P. tremuloides* clone No. CO5-99-34). The arrows indicate the beginning and the end of the heat pulse. Photosynthetic photon flux density was 600 μmol m^−2^ s^−1^.

## Abbreviations

*A*:	net CO_2_ assimilation rate
*F*_0_:	dark-adapted minimum fluorescence yield
*F*_m_:	dark-adapted maximum fluorescence yield
*F*_m_´:	light-adapted maximum fluorescence yield
*F*_s_´:	steady-state fluorescence yield
PSII:	photosystem II
*R*_w_:	stomatal resistance to water vapor;
*Φ*_PSII_:	effective PSII quantum yield

## References

[1] Holbo H.R., Childs S.W., McNabb D.H. (1985). Solar radiation at seedling sites below partial canopies. For. Ecol. Manag..

[2] Valladares F., Niinemets Ü., Pugnaire F.I., Valladares F. (2007). The architecture of plant crowns: From design rules to light capture and performance. Handbook of functional plant ecology.

[3] Porcar-Castell A., Palmroth S. (2012). Modelling photosynthesis in highly dynamic environments: The case of sunflecks. Tree Physiol..

[4] Way D.A., Pearcy R.W. (2012). Sunflecks in trees and forests: From photosynthetic physiology to global change biology. Tree Physiol..

[5] Pearcy R.W. (1990). Sunflecks and photosynthesis in plant canopies. Annu. Rev. Plant Physiol. Plant Mol. Biol..

[6] Young D.R., Smith W.K. (1979). Influence of sunflecks on the temperature and water relations of two subalpine understory congeners. Oecologia.

[7] Leakey A.D.B., Press M.C., Scholes J.D. (2003). High-temperature inhibition of photosynthesis is greater under sunflecks than uniform irradiance in a tropical rain forest tree seedling. Plant Cell Environ..

[8] Watling J.R., Robinson S.A., Woodrow I.E., Osmond C.B. (1997). Responses of rainforest understorey plants to excess light during sunflecks. Aust. J. Plant Physiol..

[9] Grace J. (1988). Plant response to wind. Agric. Ecosyst. Environ..

[10] Roden J.S., Pearcy R.W. (1993). The effect of flutter on the temperature of poplar leaves and its implications for carbon gain. Plant Cell Environ..

[11] Osmond C.B., Anderson J.M., Ball M.C., Egerton J.G., Press M.C., Scholes J.D., Barker M.G. (1999). Compromising efficiency: the molecular ecology of light-resource utilization in plants. Physiological plant ecology. The 39th Symposium of the British Ecological Society held at the University of York, 7–9 September 1998.

[12] Takahashi S., Murata N. (2008). How do environmental stresses accelerate photoinhibition?. Trends Plant Sci..

[13] Gururani M.A., Venkatesh J., Phan Tran L.-S. (2015). Regulation of photosynthesis during abiotic stress-induced photoinhibition. Mol. Plant.

[14] Niinemets Ü., Keenan T.F. (2014). Photosynthetic responses to stress in Mediterranean evergreens: Mechanisms and models. Environ. Exp. Bot..

[15] Singsaas E.L., Laporte M.M., Shi J.-Z., Monson R.K., Bowling D.R., Johnson K., Lerdau M., Jasentuliytana A., Sharkey T.D. (1999). Kinetics of leaf temperature fluctuation affect isoprene emission from red oak (*Quercus rubra*) leaves. Tree Physiol..

[16] Wise R.R., Olson A.J., Schrader S.M., Sharkey T.D. (2004). Electron transport is the functional limitation of photosynthesis in field-grown Pima cotton plants at high temperature. Plant Cell Environ..

[17] Michaletz S.T., Weiser M.D., Zhou J., Kaspari M., Helliker B.R., Enquist B.J. (2015). Plant thermoregulation: Energetics, trait–environment interactions, and carbon economics. Trends Ecol. Evol..

[18] Hamerlynck E.P., Knapp A.K. (1994). Leaf-level responses to light and temperature in two co-occurring *Quercus* (*Fagaceae*) species: Implications for tree distribution patterns. Forest Ecol. Manag..

[19] Allen C.D., Macalady A.K., Chenchouni H., Bachelet D., McDowell N., Vennetier M., Kitzberger T., Rigling A., Breshears D.D., Hogg E.H.T. (2010). A global overview of drought and heat-induced tree mortality reveals emerging climate change risks for forests. Forest Ecol. Manag..

[20] Haldimann P., Feller U. (2004). Inhibition of photosynthesis by high temperature in oak (*Quercus pubescens* L.) leaves grown under natural conditions closely correlates with a reversible heat-dependent reduction of the activation state of ribulose-1,5-bisphosphate carboxylase/oxygenase. Plant Cell Environ..

[21] Salvucci M.E., Crafts-Brandner S.J. (2004). Inhibition of photosynthesis by heat stress: The activation state of Rubisco as a limiting factor in photosynthesis. Physiol. Plant..

[22] Kim K., Portis A.R. (2005). Temperature dependence of photosynthesis in *Arabidopsis* plants with modifications in Rubisco activase and membrane fluidity. Plant Cell Physiol..

[23] Sharkey T.D. (2005). Effects of moderate heat stress on photosynthesis: Importance of thylakoid reactions, Rubisco deactivation, reactive oxygen species, and thermotolerance provided by isoprene. Plant Cell Environ..

[24] Hüve K., Bichele I., Rasulov B., Niinemets Ü. (2011). When it is too hot for photosynthesis: Heat-induced instability of photosynthesis in relation to respiratory burst, cell permeability changes and H_2_O_2_ formation. Plant Cell Environ..

[25] Zhu L., Bloomfield K.J., Hocart C.H., Egerton J.J.G., O’Sullivan O.S., Penillard A., Weerasinghe L.K., Atkin O.K. (2018). Plasticity of photosynthetic heat tolerance in plants adapted to thermally contrasting biomes. Plant Cell Environ..

[26] O’Sullivan O.S., Heskel M.A., Reich P.B., Tjoelker M.G., Weerasinghe K.W.L.K., Penillard A., Zhu L., Egerton J.J.G., Bloomfield K.J., Creek D. (2017). Thermal limits of leaf metabolism across biomes. Global Change Biol..

[27] Berry J., Björkman O. (1980). Photosynthetic response and adaptation to temperature in higher plants. Annu. Rev. Plant Physiol..

[28] Bilger H.W., Schreiber U., Lange O.L. (1984). Determination of leaf heat resistance: Comparative investigation of chlorophyll fluorescence changes and tissue necrosis methods. Oecologia.

[29] Seemann J.R., Berry J.A., Downton W.J.S. (1984). Photosynthetic response and adaptation to high temperature in desert plants. A comparison of gas exchange and fluorescence methods for studies of thermal tolerance. Plant Physiol..

[30] Schrader S.M., Wise R.R., Wacholtz W.F., Ort D.R., Sharkey T.D. (2004). Thylakoid membrane responses to moderately high leaf temperature in Pima cotton. Plant Cell Environ..

[31] Schrader S.M., Kleinbeck K.R., Sharkey T.D. (2007). Rapid heating of intact leaves reveals initial effects of stromal oxidation on photosynthesis. Plant Cell Environ..

[32] Raison J.K., Berry J.A. (1979). Viscotropic denaturation of chloroplast membranes and acclimation to temperature by adjustment of lipid viscosity. Carnegie Inst. Wash. Yearb..

[33] Smith K.A., Low P.S. (1989). Identification and partial characterization of the denaturation transition of the photosystem II reaction center of spinach chloroplast membranes. Plant Physiol..

[34] Yamane Y., Kashino Y., Koike H., Satoh K. (1998). Effects of high temperatures on the photosynthetic systems in spinach: Oxygen-evolving activities, fluorescence characteristics and the denaturation process. Photosynth. Res..

[35] Niinemets Ü. (2018). When leaves go over the thermal edge. Plant Cell Environ..

[36] Copolovici L., Kännaste A., Pazouki L., Niinemets Ü. (2012). Emissions of green leaf volatiles and terpenoids from *Solanum lycopersicum* are quantitatively related to the severity of cold and heat shock treatments. J. Plant Physiol..

[37] Tullus A., Tullus H., Vares A., Kanal A. (2007). Early growth of hybrid aspen (*Populus x wettseteinii* Hämet-Ahti) plantations on former agricultural lands in Estonia. Forest Ecol. Manag..

[38] Tullus A., Tullus H., Soo T., Pärn L. (2009). Above-ground biomass characteristics of young hybrid aspen (*Populus tremula* L. × *P. tremuloides* Michx.) plantations on former agricultural land in Estonia. Biomass Bioenergy.

[39] Niinemets Ü., Oja V., Kull O. (1999). Shape of leaf photosynthetic electron transport versus temperature response curve is not constant along canopy light gradients in temperate deciduous trees. Plant Cell Environ..

[40] Laisk A., Oja V., Rasulov B., Rämma H., Eichelmann H., Kasparova I., Pettai H., Padu E., Vapaavuori E. (2002). A computer-operated routine of gas exchange and optical measurements to diagnose photosynthetic apparatus in leaves. Plant Cell Environ..

[41] Bajji M., Kinet J.-M., Lutts S. (2002). The use of the electrolyte leakage method for assessing cell membrane stability as a water stress tolerance test in durum wheat. Plant Growth Regul..

[42] Whitlow T.H., Bassuk N.L., Ranney T.G., Reichert D.L. (1992). An improved method for using electrolyte leakage to assess membrane competence in plant tissues. Plant Physiol..

[43] Scotti Campos P., Quartin V., Cochicho Ramalho J., Nunes M.A. (2003). Electrolyte leakage and lipid degradation account for cold sensitivity in leaves of *Coffea* sp.. J. Plant Physiol..

[44] R Development Core Team (2019). R: A language and environment for statistical computing..

[45] Mulkey S.S., Pearcy R.W. (1992). Interactions between acclimation and photoinhibition of photosynthesis of a tropical forest understorey herb, *Alocasia macrorrhiza*, during simulated canopy gap formation. Funct. Ecol..

[46] Skillman J.B., Winter K. (1997). High photosynthetic capacity in a shade-tolerant Crassulacean acid metabolism plant. Implications for sunfleck use, nonphotochemical energy dissipation, and susceptibility to photoinhibition. Plant Physiol..

[47] Niinemets Ü., Kollist H., García-Plazaola J.I., Hernández A., Becerril J.M. (2003). Do the capacity and kinetics for modification of xanthophyll cycle pool size depend on growth irradiance in temperate trees?. Plant Cell Environ..

[48] Singsaas E.L., Sharkey T.D. (1998). The regulation of isoprene emission responses to rapid leaf temperature fluctuations. Plant Cell Environ..

[49] June T., Evans J.R., Farquhar G.D. (2004). A simple equation for the reversible temperature dependence of photosynthetic electron transport: A study on soybean leaf. Funct. Plant Biol..

[50] Rekika D., Kara Y., Souyris I., Nachit M.M., Asbati A., Monneveux P. (2000). The tolerance of PSII to high temperatures in durum wheat (*T. turgidum* conv. *durum*): Genetic variation and relationship with yield under heat stress. Cereal Res. Commun..

[51] Havaux M. (1993). Rapid photosynthetic adaptation to heat stress triggered in potato leaves by moderately elevated temperatures. Plant Cell Environ..

[52] Königer M., Harris G.C., Pearcy R.W. (1998). Interaction between photon flux density and elevated temperatures on photoinhibition in *Alocasia macrorrhiza*. Planta.

[53] Bilger W., Schreiber U., Lange O.L., Tenhunen J.D., Catarino F.M., Lange O.L., Oechel W.C. (1987). Chlorophyll fluorescence as an indicator of heat induced limitation of photosynthesis in *Arbutus unedo* L.. Plant response to stress. Functional analysis in Mediterranean ecosystems.

[54] Hüve K., Bichele I., Ivanova H., Keerberg O., Pärnik T., Rasulov B., Tobias M., Niinemets Ü. (2012). Temperature responses of dark respiration in relation to leaf sugar concentration. Physiol. Plant..

[55] Zhang R., Sharkey T.D. (2009). Photosynthetic electron transport and proton flux under moderate heat stress. Photosynth. Res..

[56] Zhang R., Cruz J.A., Kramer D.M., Magallanes-Lundback M.E., DellaPenna D., Sharkey T.D. (2009). Moderate heat stress reduces the pH component of the transthylakoid proton motive force in light-adapted, intact tobacco leaves. Plant Cell Environ..

[57] Eliáš P. (1979). Stomatal oscillations in adult forest trees in natural environment. Biol. Plant..

[58] Laisk A., Oja V., Walker D., Heber U. (1992). Oscillations in photosynthesis and reduction of photosystem I acceptor side in sunflower leaves. Functional cytochrome b_6_/f-photosystem I ferredoxin-NADP reductase supercomplexes. Photosynthetica.

[59] Rasulov B., Talts E., Niinemets Ü. (2016). Spectacular oscillations in plant isoprene emission under transient conditions explain the enigmatic CO_2_ response. Plant Physiol..

[60] Steppe K., Dzikiti S., Lemeur R., Milford J.R. (2006). Stomatal oscillations in orange trees under natural climatic conditions. Ann. Bot..

[61] Kask K., Kännaste A., Talts E., Copolovici L., Niinemets Ü. (2016). How specialized volatiles respond to chronic and short-term physiological and shock heat stress in *Brassica nigra*. Plant Cell Environ..

[62] Pazouki L., Kanagendran A., Li S., Kännaste A., Rajabi Memari H., Bichele R., Niinemets Ü. (2016). Mono- and sesquiterpene release from tomato (*Solanum lycopersicum*) leaves upon mild and severe heat stress and through recovery: From gene expression to emission responses. Environ. Exp. Bot..

[63] Wright I.J., Ning D., Maire V., Prentice I.C., Westoby M., Díaz S., Gallagher R.V., Jacobs B.F., Kooyman R., Law E.A. (2017). Global climatic drivers of leaf size. Science.

[64] Pshybytko N.L., Kruk J., Kabashnikova L.F., Strzalka K. (2008). Function of plastoquinone in heat stress reactions of plants. Biochim. Biophys. Acta.

[65] Laisk A., Rasulov B.H., Loreto F. (1998). Thermoinhibition of photosynthesis as analyzed by gas exchange and chlorophyll fluorescence. Russ. J. Plant Physiol..

[66] Kubien D.S., Sage R.F. (2008). The temperature response of photosynthesis in tobacco with reduced amounts of Rubisco. Plant Cell Environ..

[67] Sharkey T.D., Badger M.R., von Caemmerer S., Andrews T.J. (2001). Increased heat sensitivity of photosynthesis in tobacco plants with reduced Rubisco activase. Photosynth. Res..

[68] Tóth S.Z., Schansker G., Kissimon J., Kovács L., Garab G., Strasser R.J. (2005). Biophysical studies of photosystem II-related recovery processes after a heat pulse in barley seedlings (*Hordeum vulgare* L.). J. Plant Physiol..

[69] Havaux M., Greppin H., Strasser R.J. (1991). Functioning of photosystems I and II in pea leaves exposed to heat stress in the presence or absence of light. Analysis using *in-vivo* fluorescence, absorbance, oxygen and photoacoustic measurements. Planta.

