# Supplementary figures and images for: Responses of Aspen Leaves to Heatflecks: Both Damaging and Non-Damaging Rapid Temperature Excursions Reduce Photosynthesis
Source: Plants (Basel). 2019 May 30;8(6):145. doi: 10.3390/plants8060145 (PMC6630322; doi:10.3390/plants8060145)

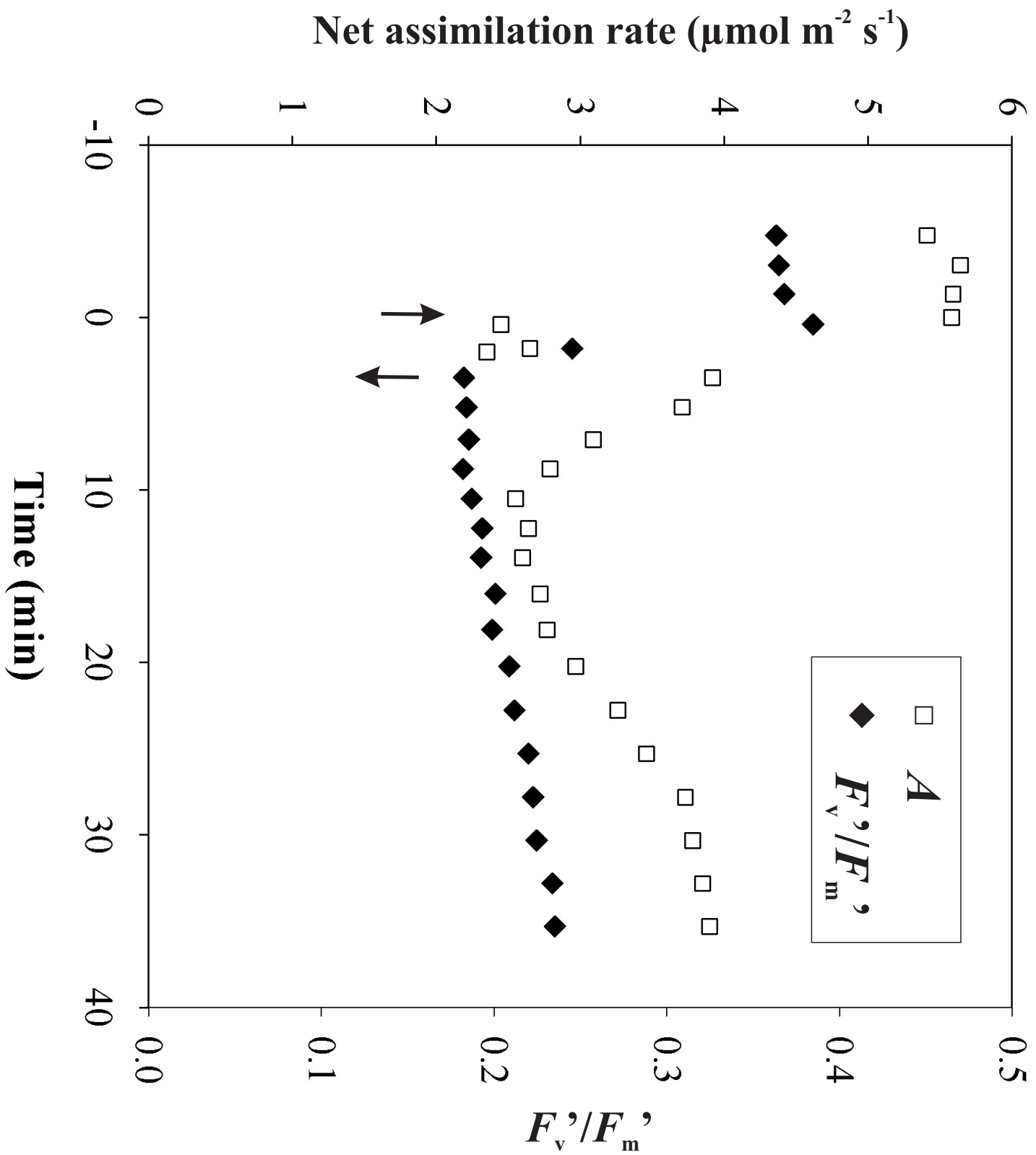

Supplement: Supplementary file 1 [file plants-08-00145-s001.pdf]
